# Triacylglycerol is produced from starch and polar lipids in the green alga *Dunaliella tertiolecta*

**DOI:** 10.1093/jxb/erx280

**Published:** 2017-09-11

**Authors:** Uri Pick, Omri Avidan

**Affiliations:** 1 Department of Biomolecular Sciences, The Weizmann Institute of Science, Rehovot, Israel; 2 Max Planck Institute of Molecular Plant Physiology, Potsdam-Golm, Germany

**Keywords:** Digalacosyldiacylglycerol, *Dunaliella tertiolecta*, fatty acids, pulse labeling, starch, triacylglycerol biosynthesis

## Abstract

The halotolerant green alga *Dunaliella tertiolecta* accumulates starch and triacylglycerol (TAG) amounting to 70% and 10–15% of total cellular carbon, respectively, when exposed to nitrogen (N) deprivation. The purpose of this study was to clarify the inter-relationships between the biosynthesis of TAG, starch, and polar lipids (PLs) in this alga. Pulse labeling with [^14^C]bicarbonate was utilized to label starch and [^14^C]palmitic acid (PlA) to label lipids. Transfer of ^14^C into TAG was measured and used to calculate rates of synthesis. About two-thirds of the carbon in TAG originates from starch, and one-third is made *de novo* by direct CO_2_ assimilation. The level made from degradation of pre-formed PLs is estimated to be very small. Most of the *de novo* synthesis involves fatty acid transfer through PLs made during the first day of N deprivation. The results suggest that starch made by photosynthetic carbon assimilation at the early stages of N deprivation is utilized for synthesis of TAG. Trans-acylation from PLs is the second major contributor to TAG biosynthesis. The utilization of starch for TAG biosynthesis may have biotechnological applications to optimize TAG biosynthesis in algae.

## Teaser

The major carbon source for triacylglycerol (TAG) biosynthesis in *Dunaliella* is starch, whose production precedes that of TAG under nitrogen deprivation.

## Introduction

**Fig. 1.**
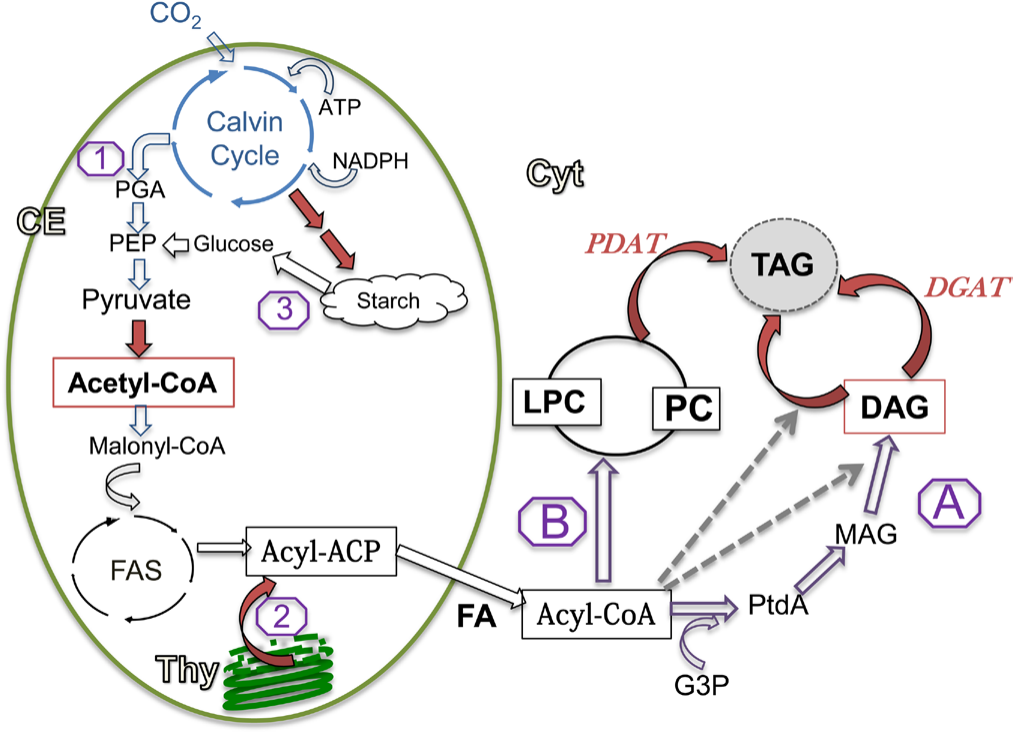
Metabolic pathways for TAG biosynthesis in green algae. FA synthesis pathways: (1) integration of newly assimilated carbon; (2) degradation of pre-formed polar lipids. (3) degradation of starch. Enzymes and substrates that are up-regulated under N deprivation are marked in italics. TAG synthesis pathways: (A) the Kennedy pathway, (B) PC acyl editing. CE, chloroplast envelope; Cyt, cytoplasm; FAS, fatty acid synthase pathway; ACP, acyl-carrier protein; PEP, phosphoenol pyruvate; PGA, 3-phosphoglyceric acid; G3P, glycerol-3-phosphate; PtdA, phosphatidic acid; MAG, monoacylglycerol; DAG, diacylglycerol; LPC, lyso-phosphatidylcholine; PDAT, phospholipid diacylglycerol acyltransferase; DGAT, diacylglycerol acyltransferase. (This figure is available in colour at *JXB* online.)

Under stress conditions such as nitrogen (N) deprivation, green microalgae synthesize large amounts of starch and/or of triacylglycerol (TAG) (Razeghifard, 2013; Slocombe *et al.*, 2015). The exact metabolic pathways leading to TAG biosynthesis are not clear. Lipid biosynthesis in higher plants can be divided into two stages: fatty acid (FA) biosynthesis, which takes place in the chloroplast, and assimilation of the acyl chains into the glycerol backbone to form TAG which takes place in the endoplasmic reticulum (ER) (Bates *et al.*, 2009; Bates, 2016). Recent studies indicate that in eukaryotic algae the process may be more complicated and involve more diverse metabolic pathways. In some algae such as in *Chlamydomonas reinhardtii*, it was shown that TAG biosynthesis also takes place inside the chloroplast (Fan *et al.*, 2011; Goodson *et al.*, 2011). At least three different metabolic pathways may allocate carbon to FA biosynthesis in the chloroplast of green algae (Goncalves *et al*, 2016): (i) *de novo* synthesis directly from CO_2_ assimilation by the Calvin cycle, formation of acetyl-CoA by pyruvate dehydrogenase, and its assimilation via the fatty acid synthesis (FAS) cycle (pathway 1; Fig. 1); (ii) FA acyl transfer from pre-formed polar lipids (PLs), particularly of chloroplast galactoglycerolipids (pathway 2; Fig. 1); phytylester synthases (PESs) may be good candidates to catalyze this reaction (Lippold *et al.*, 2012); and (iii) synthesis from degradation of pre-formed starch (pathway 3; Fig. 1). The subsequent incorporation of acyl groups into glycerol may take place either by three consecutive incorporations from acyl-CoA into glycerol-3-phosphate, known as the Kennedy pathway (pathway A; Fig. 1; Ohlrogge and Browse, 1995; Colemann and Lee, 2004), or via PLs, such as phosphatidylcholine (PC), followed by acyl transfer into TAG, also termed ‘acyl editing’ (pathway B; Fig. 1, Harwood, 1996; Bates *et al.*, 2009, Bates, 2016). However, hardly any direct quantitative measurements have been made to test how much each pathway contributes to the overall TAG biosynthesis.

The inter-relationships between starch and TAG biosynthesis are not clear. In several algae species which accumulate both starch and TAG, and also in developing oilseed rape embryos, starch precedes TAG biosynthesis and in some species the level of starch decreases in parallel with TAG accumulation, suggesting that the latter may be synthesized from degradation of the former (Eastmond and Rawsthorne, 2000; Siaut *et al.*, 2011; Longworth *et al.*, 2012, 2016; Recht *et al.*, 2012, 2014). Transcriptome and lipidome analyses performed in *Dunaliella tertiolecta* and in *Nannochloropsis* have shown that N deprivation induces degradation of carbohydrate that seems to be temporally co-ordinated with mass biosynthesis of neutral lipids. The authors suggested that these findings represent a carbon flow from the former to the latter (Rismani-Yazdi *et al.*, 2011; Li *et al.*, 2014). A recent study in *Chlamydomonas reinhardtii* shows that whereas most starch is made from assimilated CO_2_, most FAs and TAG are produced from acetate (Juergens *et al.*, 2016). These studies are consistent with two parallel biosynthetic mechanisms, one for starch and another for TAG.

*Dunaliella tertiolecta* is a promising organism for industrial utilization for production of biofuels for several reasons: it is a fast-growing species and one of the top biomass produces; it produces very high concentrations of starch amounting to >60% of its dry weight and also moderate levels of lipids (Tang *et al.*, 2011; Slocombe *et al.*, 2015); and it is a robust species that maintains high growth rates at extreme pH, temperature, and salt concentrations. In fact, it is model salt-tolerant organism, since it can adapt to an exceptionally wide range of NaCl concentrations from 50 mM up to saturated salt solutions (Katz *et al.*, 2009). This enabled outdoor cultivation at high NaCl concentrations, which minimizes the danger of contamination, a major risk factor in cultivation of microalgae. A special feature of this species is that it enhances photosynthetic CO_2_ fixation and starch accumulation at high salinity by up-regulation of the Calvin cycle enzymes, thus maintaining high biomass productivity also at high NaCl concentrations (Liska *et al.*, 2004). *Dunaliella tertiolecta*, like other *Dunaliella* species, lacks a cell wall, enabling easy cell lysis, which lowers the cost of processing of this alga. Two major drawbacks for molecular studies in this species are that it has not yet been sequenced and that its transformation is not straightforward even though several successful reports of nuclear and chloroplast transformations have been reported (Georgianna *et al.*, 2013; Zhang *et al.*, 2015). Recently, several new studies were reported which provide advanced tools and databases for molecular analysis of *D. tertiolecta*: A preliminary annotation of *D. tertiolecta* genes, based on sequence similarity to other green algae, was reported in 2011 (Rismani-Yazdi *et al.*, 2011). In 2015, a draft database for annotation of RNA-seq analysis was constructed and utilized to analyze metabolic pathways that are enhanced in a high neutral lipid-producing mutant (Yao *et al.*, 2015), and a similar transcriptome annotation of *D. tertiolecta* was constructed and utilized to analyze changes in gene expression during N deprivation (Shin *et al.*, 2015). In 2016, a more comprehensive transcriptome analysis of *D. tertiolecta* was conducted via RNA-seq with the aim to clarify the metabolic changes involved in starch and neutral lipid biosynthesis during N deprivation (Tan *et al.*, 2016). The work uncovered up-regulation of central metabolic pathways leading to starch accumulation and minor or no up-regulation of lipid-metabolizing enzymes, leading to the conclusion that lipids are not a preferred storage product in *D. tertiolecta*.

However, none of these studies referred to interconvertions between starch and lipids or between polar and neutral lipids and their potential contributions to TAG formation.

In order to try to clarify the metabolic inter-relationships between PLs, starch, and TAG biosynthesis, we designed a set of pulse labeling experiments using ^14^C to measure the carbon flow from PLs and starch into TAG. The experiments were carried out in *D. tertiolecta*, which is particularly suitable for such a study because when grown in the absence of N, starch accumulation precedes TAG biosynthesis by several days (see also Tan *et al.*, 2016), thus enabling convenient measurement of conversion of starch carbon into TAG. In order to measure the carbon flux from starch into TAG, cells were pulse-labeled with [^14^C]bicarbonate at the early stages of N deprivation, and the fate of ^14^C in TAG and starch was followed. In order to measure how much TAG is produced from FAs released from degradation of PLs, we pre-labeled PLs with ^[4^C]palmitic acid (PlA) before the onset of N deprivation and followed the fate of PlA in TAG and in PLs. Our results show that under N deprivation, the major carbon source for TAG biosynthesis is starch; pre-formed PLs contribute relatively little carbon, and a significant amount of TAG is made by *de novo* synthesis through newly formed PLs.

## Materials and methods

### Radioactive chemicals

[^14^C]Sodium bicarbonate (NEC086HOO, 53 mCi mmol^–1^) and [^14^C]PlA (NEC075HO, 60 mCi mmol^–1^) were purchased from PerkinElmer, Waltham, MA, USA.

### Algal strains and cultivation conditions

*Dunaliella tertiolecta* was obtained from the culture collection of Dr W.H. Thomas (La Jolla, CA, USA). Cells were cultured under continuous illumination (at a light intensity of 120–150 μmol m^−2^ s^−1^) on a shaker set at 100 rpm at 24 °C, as previously described (Ben-Amotz *et al.*, 1989). The media were supplemented with 0.5 M NaCl and 50 mM sodium bicarbonate. To induce N limitation, mid-log phase cells (cultured for 48 h) were centrifuged for 5 min at 5000 *g*, washed once with growth medium lacking KNO_3_ (–N medium), and suspended in –N medium. Cells were counted with the Cellometer automated cell counter (Nexelom Bioscience LLC, Lawrence, MA, USA).

### Labeling with [^14^C]bicarbonate

*Dunaliella tertiolecta* cells were labeled with 50 mM [^14^C]bicabonate (0.16 μCi ml^–1^) in growth media containing 5 mM K-nitrate (+N) or no nitrate (–N). For photosynthetic CO_2_ uptake activity, cells were incubated for 1 h in an illuminated shaker at 24 °C. This uptake activity is completely light dependent, and >98% of the ^14^C taken up by the cells is assimilated (not releasable by acid treatment). Therefore, this uptake activity represents photosynthetic CO_2_ assimilation. Washed cell pellets were dissolved in 0.2% Triton X-100 and 20% sodium hypochlorite (bleach) to quench pigments before radioactivity counting. For total carbon labeling that was used to calculate starch and TAG contents, cells were diluted to 3 × 10^5^ cells ml^–1^ in complete growth medium containing 50 mM [^14^C]bicabonate and incubated for 48 h in the light. At the end of the incubation, the cell concentration was 1.5–2.2 × 10^7^ cells ml^–1^. The cells were washed once and diluted 2-fold into N-depleted medium containing the same concentration of [^14^C]bicabonate and incubated under continuous illumination for 8 d. For pulse labeling, cells were incubated with [^14^C]bicarbonate for 24 h as indicated in each experiment. At the end of the incubations, cells were washed twice in fresh medium containing 50 mM bicarbonate to remove traces of [^14^C[bicarbonate and either frozen and kept at –20 °C for lipid extraction or suspended in fresh growth medium for continued culturing (pulse labeling). All samples were counted in a scintillation β-counter with 10 ml of scintillation cocktail (Ultima Gold, Perkin Elmer).

### Pulse labeling with [^14^C]PlA

#### Pre-labeling membrane polar lipids in complete (+N) growth medium

*Dunaliella tertiolecta* cells cultured in complete growth medium (+N) were pulse-labeled for 2 h with [^14^C]PlA (1–2.5 μCi 100 ml^–1^, 0.5 μM) in complete growth medium. Cells were then washed once in –N medium in the presence of 100 μM PlA, then incubated for 10–20 min in –N growth medium containing 1% defatted BSA to eliminate all traces of [^14^C]PlA, washed once again in –N growth medium, and finally suspended in fresh –N growth medium for continued incubation in the light. Samples were removed immediately and at daily intervals, pelleted by centrifugation, and stored at –20 °C.

#### Labeling during the first day of N deprivation

The growth conditions were as above, except that labeling with [^14^C]PlA was performed during the first 24 h of N deprivation.

#### Lipid *de novo* synthesis from [^14^C]PlA during N deprivation

This experiment was designed to estimate how much [^14^C]PlA is incorporated into PLs and into TAG during each day of N deprivation. Cell samples were labeled for 24 h with [^14^C]PlA during days 1–8 of N deprivation as described above.

### Lipid extraction and cell fractionation

Lipids were extracted essentially as described before (Avidan *et al.*, 2015). Cell pellets were extracted in MeOH:CHCl_3_:H_2_O, 1:1:1, and separated into starch pellets, the water–methanol phase, and the chloroform lipid phase. The water–methanol phase was re-extracted once with chloroform to remove residual lipids and kept as the water-soluble fraction. The chloroform lipid phase was dried under reduced pressure and dissolved in 150 μl of CHCl_3_ (total lipids fraction), and kept at –20 °C for lipid analysis by TLC.

### Determination of starch

The pellets remaining after the extraction of lipids, which contain starch and proteins, were washed once in cold methanol to remove residual lipids and incubated in 1 ml of 0.2% Triton X-100, 20 mM Tris–HCl, pH 7.5 solution containing 10 μg of trypsin (sequencing grade modified porcine trypsin, Promega, Madison, WI, USA) for 6 h at 25 °C to degrade proteins, followed by one wash in Triton X-100 buffer. ^14^C-Labeled samples were counted in a scintillation β-counter with 10 ml of scintillation cocktail (Ultima Gold, Perkin Elmer). Starch was also determined independently by the iodine method as previously described (Avidan *et al.*, 2015).

### Separation of lipids on TLC plates

Lipid samples were separated on TLC Silica gel 60 aluminum sheets (Merck, Darmstadt, Germany). Neutral lipids were separated in *n*-hexane:diethylether:acetic acid (85:22:1). PLs were separated either in chloroform:methanol:acetic acid:water (90:20:12:4) or in chloroform:methanol:water (60:30:4). Lipid spots were detected by iodine staining and quantified by densitometric analysis with reference to triolein standards of 0.5–2 µg as previously described (Davidi *et al.*, 2015). Identification of specific lipid bands was verified by comparison to lipid standards, by staining for phospholipids, galactoglycerolipids, or PC, and by MS. TAG, FAs, and diacylglycerol (DAG) were resolved as shown in Supplementary Fig. S1A at *JXB* online; monogalactosyl diacylglycerol (MGDG), digalactosyl diacylglycerol (DGDG), and sulfoquinovosyl diacylglycerol (SQDG) as in Supplementary Fig. S1C; and PC and phosphatidylglycerol (PG) as in Supplementary Fig. S1D. DAG, PC, PG, oleic acid, and a mixture of chloroplast membrane lipids (MGDG, DGDG, SQDG, and PG) were used as standards.

For determination of ^14^C contents, plates were dried, stained with iodine vapor, and the desired bands were cut out, placed in a scintillation vial, supplemented with 1 ml of CHCl_3_:MeOH, 1:1, and extracted on a shaker for 4–12 h. The extracts were transferred to a clean scintillation vial, supplemented with scintillation cocktail, and counted in a scintillation β-counter.

### Calculations

#### Total starch

The total carbon content in starch, in units of nmol C 10^6^ cells^–1^, was calculated from the ^14^C counts in the isolated starch fraction and from the [^14^C]bicarbonate specific activity (^14^C dpm μmol^–1^ bicarbonate) that was used for labeling the cells in complete growth medium for 48 h (Fig. 1; Supplementary Table S1). The starch content, in units of μg starch 10^6^ cells^–1^ (Supplementary Table S2), was calculated from the carbon content by assuming that carbon (^14^C) consists of 40% of the total weight of starch.

#### Total TAG

The total carbon content in TAG, in units of nmol C 10^6^ cells^−1^, was calculated from the ^14^C counts in the isolated TAG and from the [^14^C]bicarbonate specific activity as for starch (Fig. 1; Supplementary Table S1). The TAG contents, in units of μg TAG 10^6^ cells^−1^ (Supplementary Table S2), was calculated from these values by assuming that carbon (^14^C) consists of 80% of the total weight of TAG.

## Results

### Starch and TAG accumulation under N deprivation in *D. tertiolecta*

**Fig. 2.**
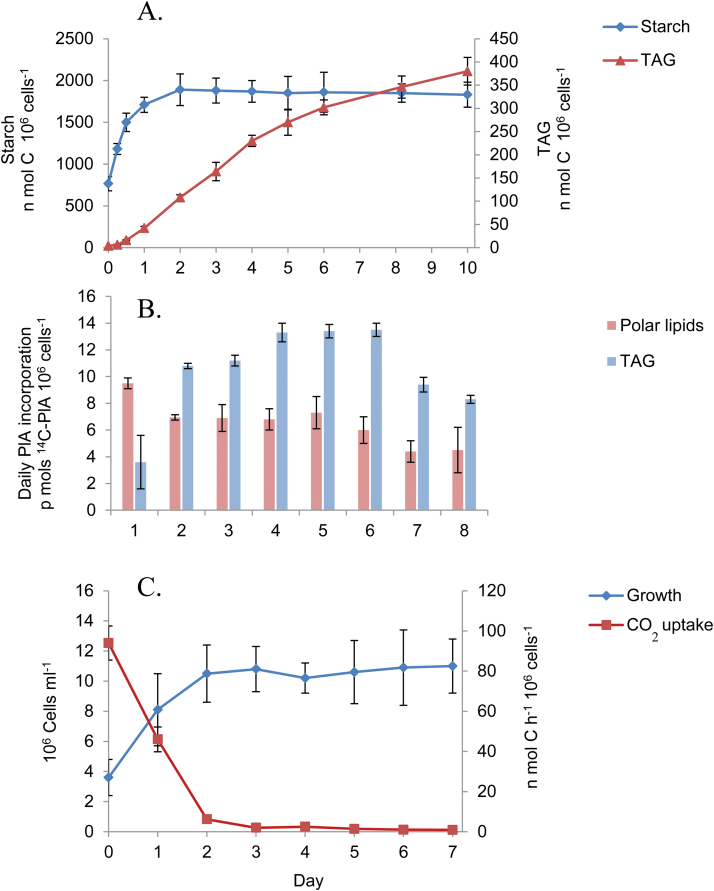
Changes in the total carbon of starch and of TAG, in cell number, and in CO_2_ assimilation activity during N deprivation. (A) Time-course of starch and TAG formation. *Dunaliella tertiolecta* cells were pre-labeled for 48 h in complete growth medium with 50 mM [^14^C]bicarbonate and then transferred to N-deficient medium containing the same [^14^C]bicarbonate concentration for 8 d. Samples were withdrawn at the indicated times, lipids were extracted and separated, and the ^14^C contents in starch and in TAG were determined. Results are expressed in nmol C 10^6^ cells^−1^ (means of three independent experiments). (B). Daily changes in *de novo* synthesis activity of TAG and of PLs during the progress of N deprivation. *Dunaliella tertiolecta* cells were pulse-labeled for 24 h with [^14^C]PlA during each day of 8 d of N deprivation. Lipids were extracted, separated, and analyzed for ^14^C content as described in the Materials and methods (means ± SD of three independent experiments). (C). Cell proliferation and changes in photosynthetic CO_2_ assimilation. *Dunaliella tertiolecta* cells were cultured in N-deprived growth medium for 7 d. Each day samples were taken to determine cell number and CO_2_ uptake activity as described in the Materials and methods (means ± SD of three independent experiments). (This figure is available in colour at *JXB* online.)

In order to obtain an accurate measure of the levels of TAG and of starch accumulated under N deprivation, we performed a complete labeling of cells with [^14^C]bicarbonate and followed the changes in the level of ^14^C incorporated into starch and into TAG during N deprivation (Fig. 2A; Supplementary Table S1). During the 48 h of pre-incubation with 50 mM [^14^C]bicarbonate in complete growth medium, >98% of the carbon in the cells was labeled with ^14^C. Therefore, the ^14^C counts in starch and in TAG can be used to calculate their levels in the cells. The level of starch increased during 8 d without N from 765 ± 85 nmol C 10^6^ cells^−1^ to 1850 ± 110 nmol C 10^6^ cells^−1^. Assuming that carbon constitutes 40% of the weight of starch, these values correspond to 23 μg starch 10^6^ cells^−1^ and to 55.5 μg starch 10^6^ cells^–1^ in complete and N-deficient media, respectively. These values are in good agreement with starch levels of 20 ± 6 μg starch 10^6^ cells^−1^ and 70 ± 16 μg starch 10^6^ cells^−1^ that were determined in parallel experiments in complete and N-deprived growth media, respectively, by the iodine method (Supplementary Table S2; Avidan *et al.*, 2015). The major increase in starch takes place during the first 24 h without N. TAG levels increased during the same period from 4 ± 0.2 nmol C 10^6^ cells^−1^ to 346 ± 12 nmol C 10^6^ cells^−1^, which correspond to 0.08 μg TAG 10^6^ cells^−1^ and 5.6 μg TAG 10^6^ cells^−1^ for cells in complete and in N-deprived media, respectively. These values are in fairly good agreement with TAG levels determined in parallel experiments by GC analysis of FA contents of 0.3 ± 0.15 μg TAG 10^6^ cells^−1^ and 9.4 ± 1.8 μg TAG 10^6^ cells^−1^ (Supplementary Table S2). However, in contrast to starch, the production of TAG starts after a lag period of ~12 h, and accumulates mostly between days 2 and 5 of N deprivation, reaching a maximal level of ~12% of total cell carbon after 10 d without N.

To confirm that there is indeed a lag in the onset of TAG biosynthesis, we performed a complementary experiment aimed to measure TAG biosynthesis activity during the progress of N deprivation. For this, we chose to measure the incorporation of PlA, which is rapidly and specifically incorporated into all glycerolipids in *Dunaliella* (Davidi *et al.*, 2014; Supplementary Fig. S2). The estimated internal concentration of PlA is ~1 μM (Supplementary Table S3 and additional information therein), which makes it very unlikely that internal pools of PlA delay its incorporation into lipids. Cultures were incubated for 24 h with [^14^C]PlA during each day of N deprivation, from day 1 to day 8, and the level of PlA incorporated into PLs and into TAG was determined. The incorporation of PlA into TAG during the first day without N is indeed slow, reaches a maximum during the second to fifth days, and thereafter decreases (Fig. 2B), consistent with the increase in the level of TAG. These results indicate that FA incorporation into TAG is indeed delayed for 12–24 h from the onset of N deprivation. PL biosynthesis during this period slightly decreases initially and then stabilizes at approximately half of the rate of TAG biosynthesis.

In order to assess the contribution of photosynthesis to starch and TAG accumulation, we followed the changes in photosynthetic CO_2_ assimilation activity during N deprivation. The cells continue to divide during the first 2 d, resulting in a 2- to 3-fold increase in cell concentration. Photosynthetic carbon assimilation activity drops precipitously by 50% in the first day and to <10% after 2 d of N deprivation (Fig. 2C), consistent with our previous results (Avidan and Pick, 2015). This suppression of photosynthesis is probably part of the N-sparing mechanism that has been described in *Chlamydomonas* (Schmollinger *et al.*, 2014). The decrease in CO_2_ assimilation activity closely parallels the saturation in starch accumulation level, consistent with the idea that most assimilated CO_2_ is channeled into starch biosynthesis. In contrast, the major accumulation of TAG takes place when the photosynthetic carbon assimilation rate is already very low, suggesting that the major carbon source for TAG biosynthesis should come from pre-formed carbon sources, the best candidates being PLs or starch.

### Formation of TAG from starch

In order to test if starch carbon is utilized for biosynthesis of TAG, cell cultures were pulse-labeled for 24 h with [^14^C]bicarbonate during the first day of N deprivation, then the labeling medium was replaced, and the levels of ^14^C in starch, in total lipids, and in TAG were measured every day, during 14 d of N deprivation. If indeed starch is degraded and converted into FAs that are next incorporated into TAG, then it is expected that assimilated ^14^C levels will decrease in starch and appear in TAG.

**Fig. 3.**
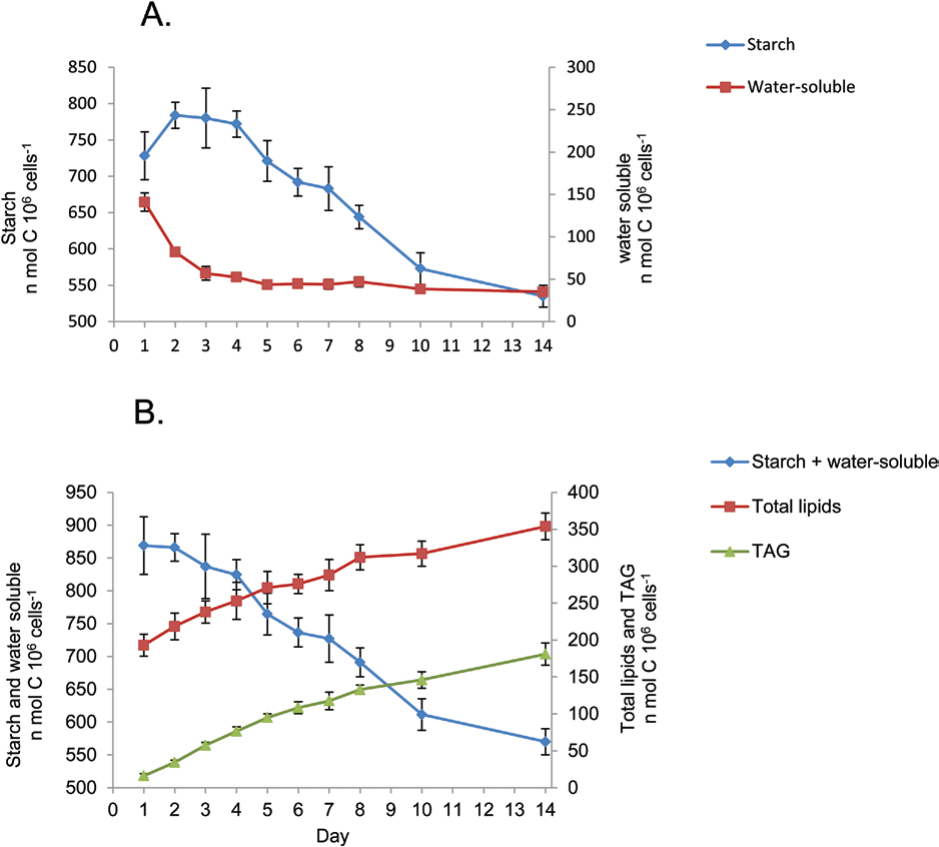
Transfer of ^14^C from starch to TAG during N deprivation*. Dunaliella tertiolecta* was cultured in N-deprived medium for 14 d. During the first 24 h, cells were labeled with [^14^C]bicarbonate. Then cells were washed twice and the medium was replaced by a fresh –N medium containing 50 mM bicarbonate. Each day samples of cells were extracted and fractionated to determine the ^14^C level in starch, polar lipids, TAG, and in the water-soluble fraction, as described in the Materials and methods. (A) Changes in ^14^C levels in starch and in the water-soluble fraction. (B) Changes in ^14^C levels in the starch plus the water-soluble fraction, in the total lipid fraction, and in TAG. Results are expressed in nmol C 10^6^ cells^−1^ (means ± SD of three independent experiments). (This figure is available in colour at *JXB* online.)

As shown in Fig. 3A, the ^14^C level in starch actually increases during the second day, even though [^14^C]bicarbonate has been chased out and replaced by 50 mM [^12^C]bicarbonate. In parallel to the increase in starch ^14^C, there is a steep decrease in the ^14^C level in the water-soluble fraction, which contains mostly glycerol, sugar phosphates, and amino acids (Ben-Amotz and Avron, 1973). It appears, therefore, that the increase in ^14^C level in starch results from conversion and assimilation of soluble carbohydrates and/or glycerol into starch.

Plotting the sum of ^14^C in starch and the soluble fraction against time of N deprivation shows that there is indeed a decrease in the ^14^C content in starch with a parallel increase in ^14^C content in TAG, starting from the second day of N deprivation (Fig. 3B). The total increase in TAG (180 nmol C 10^6^ cells^−1^) amounts to 60% of the decrease in starch (300 nmol C 10^6^ cells^−1^). Comparison of the changes in the total level of starch (Fig. 2A) with the level of starch formed during the first day of N deprivation (Fig. 3) shows that whereas the total starch level reaches a steady state after 48 h, the starch formed during the first day continuously decreases. This indicates that starch biosynthesis continues at a reduced rate during N deprivation in parallel with the conversion of part of the starch into TAG, keeping a fairly constant steady-state level of starch.

**Fig. 4.**
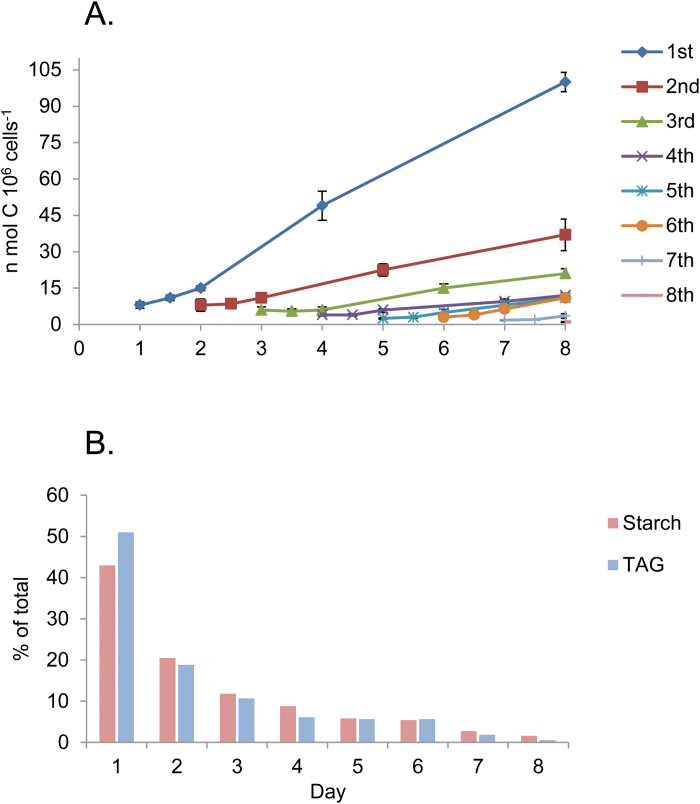
Transfer of ^14^C from starch made during different days of N deprivation into TAG. (A) Changes in ^14^C levels in TAG. Parallel cultures of *D. tertiolecta* were pulse-labeled for 24 h with [^14^C]bicarbonate during different days of N deprivation. At the end of labeling, cells were washed and transferred to fresh N-deprived media containing 50 mM sodium bicarbonate. Samples were taken for lipid extraction and fractionation at the end of labeling and at the indicated times. The ^14^C levels in starch and TAG were determined as described in the Materials and methods. (B) Fractional daily production of starch and of the total TAG produced from starch until the eighth day, expressed as a percentage of the total (data taken from A). Total levels of starch and of TAG produced in 8 d are 1453 nmol C 10^6^ cells^−1^ and 196.5 nmol C 10^6^ cells^−1^ for starch and TAG, respectively (means ± SD of three independent experiments). (This figure is available in colour at *JXB* online.)

In order to estimate how much of the starch formed during each day of N deprivation is converted to TAG, we designed a similar experiment in which cells were pulse-labeled daily for 24 h with [^14^C]bicarbonate during 8 d of N deprivation. Then the [^14^C]bicarbonate was replaced by fresh medium and the fate of assimilated ^14^C in starch and in TAG was determined immediately after removal of the label and after different times during the continued N deprivation. The results of this experiment are summarized in Fig. 4.

Most of the TAG that is produced from starch is made from starch that has been synthesized during the first day of N deprivation. It amounts to close to 50% of the total TAG produced from starch during 8 d (Fig. 4A, B). The decrease in daily TAG production from starch is proportional to the relative level of starch produced each day (Fig. 4B). Most of the TAG is produced after a 24 h delay from the end of the pulse–chase.

### *De novo* TAG biosynthesis

**Fig. 5.**
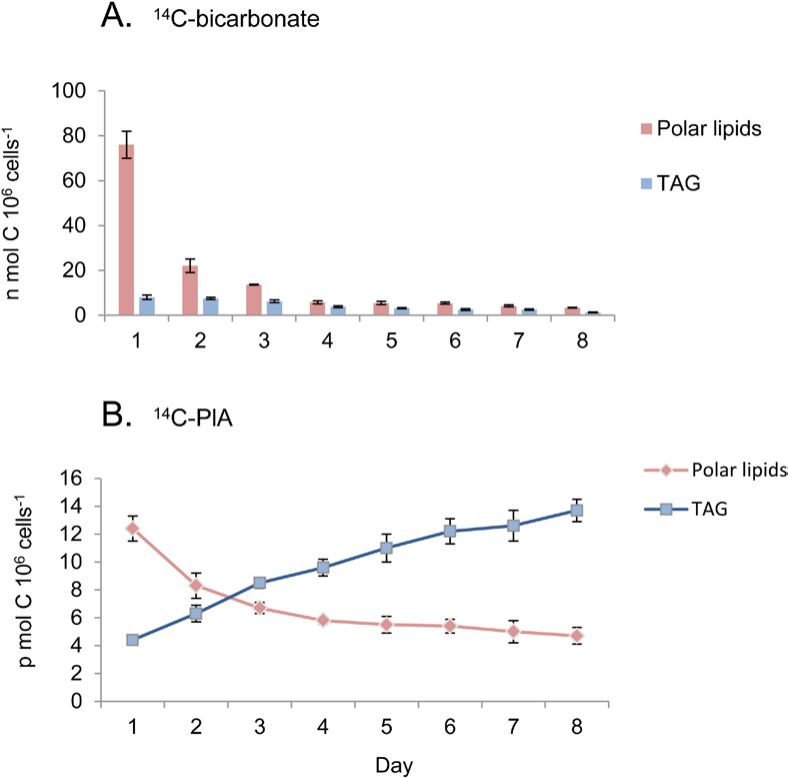
*De novo* TAG biosynthesis by direct bicarbonate incorporation and via PLs. (A) *De novo* synthesis of PLs and of TAG during each day of N deprivation. Parallel cultures of *D. tertiolecta* were pulse-labeled for 24 h with [^14^C]bicarbonate during different days of N deprivation as described in Fig. 4. At the end of labeling, cells were washed, lipids were extracted and fractionated to PLs and TAG, and the level of ^14^C in each fraction was determined and expressed in nmol C 10^6^ cells^−1^ (means ±SD of three independent experiments). (B) Formation of TAG from PLs produced during the first day of N deprivation*. Dunaliella tertiolecta* cells were labeled with [^14^C]PlA during the first 24 h of N deprivation. The cells were washed and transferred to fresh N-deprived medium. Each day samples were fractionated and analyzed. Results are expressed in pmol PlA incorporated per day per 10^6^ cells (means ± SD of three independent experiments). (This figure is available in colour at *JXB* online.)

To measure how much TAG is produced *de novo*, by direct CO_2_ assimilation, we measured the incorporation of [^14^C]bicarbonate into PLs and into TAG during each day of N deprivation. As shown in Fig. 5A, a significant amount of PLs (135 nmol C 10^6^ cells^−1^) is made *de novo*, most of it during the first day. The amounts of TAG produced *de novo* are much smaller (35 C nmol 10^6^ cells^−1^).

In order to test if part of the PLs that are formed during N deprivation are utilized for production of TAG, we designed a pulse labeling experiment with [^14^C]PlA, in which cultures were incubated with [^14^C]PlA during the first day of N deprivation, the label was removed, and the cells continued to grow without N for 7 d. Cell samples were collected daily, and lipids were extracted, separated, and counted to evaluate the level of ^14^C in PL and in TAG.

**Fig. 6.**
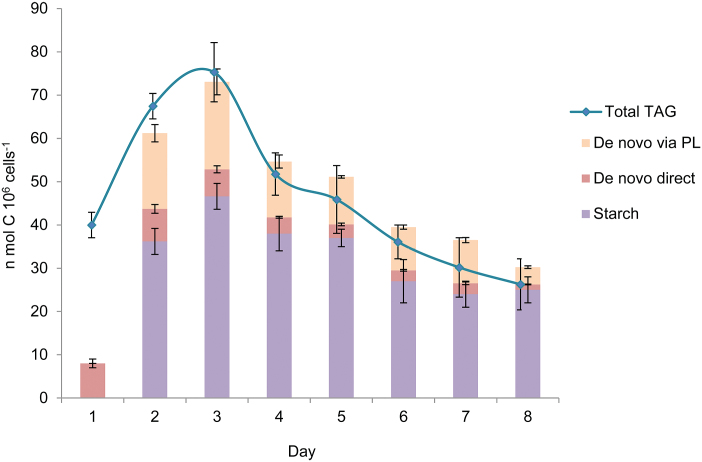
Daily production of TAG from starch, by *de novo* synthesis, and total TAG. Direct *de novo* synthesis (pink) from direct incorporation of CO_2_ was measured as described in Fig. 5A. *De novo* synthesis via newly formed PLs (orange) was determined by multiplying the levels of PLs formed during days 1–7 (Fig. 5A) by the fraction of PlA transferred each day from PLs into TAG (Fig. 5B). Synthesis from starch (marked purple) was calculated from the sums of ^14^C transferred each day from starch made during days 1–8 (Figs 3, 4). The total TAG level was calculated from the daily increase in ^14^C levels in starch in cells with total [^14^C]carbon labeling as described in Fig. 2. The results are expressed in nmol C 10^6^ cells^−1^.

As shown in Fig. 5B, over half of the [^14^C]PlA incorporated into the newly made PLs disappears during the following 2 d of N deprivation in parallel with an increase in ^14^C contents in TAG, suggesting that ~70% of the PlA in *de novo* formed PLs is channeled into TAG. Assuming that 70% of the total FAs in the PLs made during N deprivation are utilized for production of TAG enables calculation of how much TAG is produced from *de novo* made PLs. Multiplying the total PLs made during 8 d of N deprivation by 70% yields a value of 85.6 nmol C 10^6^ cells^−1^, in comparison with a total of 35 nmol TAG C 10^6^ cells^−1^ produced by direct *de novo* incorporation of bicarbonate (Fig. 6; Table 1). Comparing these values with the total level of TAG made from starch during 8 d of N deprivation (Fig. 6; Table 1), of 234 nmol C 10^6^ cell^−1^, suggests that about two-thirds of TAG is made from starch and about one-third is made *de novo*.

**Table 1.** Daily production of TAG from starch by *de novo* synthesis and total TAG

Day	Starch	*De novo* direct	*De novo* via PLs	Total calculated	Total measured
1	0	**8.0 ± 1**	0	8.0 ± 1	38 ± **3**
2	**36.2 ± 3.1**	**7.5 ± 1**	**17.5 ± 2.0**	**61.2 ± 6.1**	**66 ± 3**
3	**46.6 ± 3.3**	**6.25 ± 0.8**	**20.2 ± 2.8**	**73.1 ± 6.9**	**74 ± 7**
4	**38.0 ± 3.9**	3.75 ± 0.2	12.9 ± 1.5	54.7 ± 5.6	50 ± 5
5	**37.2 ± 2.2**	3.1 ± 0.3	11.0 ± 1.0	51.3 ± 3.5	44 ± 8
6	27.3 ± 5.1	2.5 ± 0.2	10.1 ± 0.8	39.9 ± 6.1	34 ± 4
7	24.0 ± 3.2	2.5 ± 0.3	10.0 ± 1.1	36.5 ± 4.6	28 ± 7
8	25.2 ± 3.0	1.25 ± 0.1	4.2 ± 0.5	30.6 ± 3.6	24 ± 6
Total	233.8	34.9	85.6	354.3	358

The values of TAG biosynthesis by different metabolic pathways are as described in Fig. 6. All values are expressed in nmol C 10^6^ cells^−1^. Peak values are emphasized in bold.

In order to check how these calculated values correlate with the total level of TAG in the cells, we performed a total labeling of carbon in the cells with [^14^C]bicarbonate, as described in Fig. 2, and calculated how much TAG is produced each day from the daily increase in ^14^C label in TAG. As shown in Fig. 6, the calculated sums of TAG made *de novo* and from starch each day are quite similar to the daily increase in total TAG level, except for the first day, in which the calculated sum is much lower than the total TAG. This difference results from the experimental design, which does not measure the TAG made from starch and from *de novo* made PLs during the first day of N deprivation. Pre-formed starch, made before the onset of N deprivation, may significantly contribute to the TAG produced during the first day and also during subsequent days. Also PLs that are produced during the first day may be rapidly converted into TAG already during the first day and contribute to the total TAG. Thus, both pre-formed starch and *de novo* made PLs can significantly contribute to TAG formation on the first day of N deprivation.

### Inhibition of lipid *de novo* synthesis during N deprivation

The steep decrease in incorporation of bicarbonate into PLs during the progress of N deprivation (Fig. 5A) suggests strong inhibition of *de novo* synthesis of all PLs. In order to estimate the inhibition in synthesis of different lipids, we compared the level of *de novo* synthesis of different lipids in control cells, cultured in N-replete medium, and in cultures deprived of N for 3 d. The third day was chosen because at this time point photosynthesis reaches a low steady-state value, starch accumulation levels off (Fig. 2), and the cells enter a new metabolic phase. Cultures were labeled for 24 h with [^14^C]bicarbonate under identical conditions (except for the presence or absence of nitrate), lipids were extracted and separated, and the ^14^C content was determined.

**Fig. 7.**
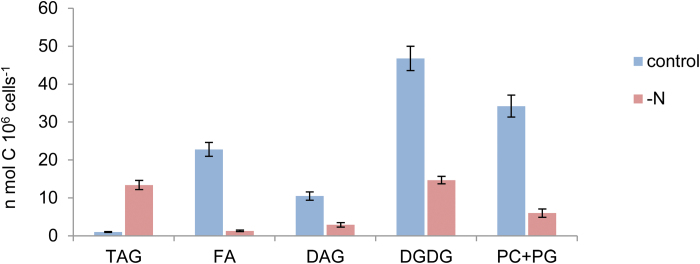
A comparison of *de novo* synthesis of different lipids between control and N-deprived *cells*. *Dunaliella tertiolecta* cells pre-cultured in complete (+N) medium or in N-deprived medium for 2 d, were pulse-labeled for 24 h with [^14^C]bicarbonate. Cells were washed, lipids were extracted and separated, and the^14^C content of each component was determined as described in the Materials and methods. (This figure is available in colour at *JXB* online.)

The comparison shows a major decrease in incorporation of bicarbonate into all lipid components, except for TAG, under N deprivation compared with control cells (Fig. 7). The decrease in assimilation into FAs is most prominent compared with all other lipid components, amounting to almost 95%, whereas the highest relative incorporation was obtained in DGDG, indicating that there is hardly any *de novo* synthesis of FAs during N deprivation, whereas about 25% of the DGDG is synthesized *de novo*.

### Formation of TAG from pre-formed PLs

Previous experiments including our own have indicated that during N deprivation considerable degradation of membrane lipids takes place, particularly of galactoglycerolipids in the chloroplast, and that part of the released FAs is incorporated into TAG (Li *et al.*, 2012; Goncalves *et al.*, 2013; Kim *et al.*, 2013; Davidi *et al.*, 2014). In fact, it is generally assumed that most polyunsaturated FAs (PUFAs) in TAG, such as 18:3 and 16:4, originate from FAs released during degradation of chloroplast membrane lipids, particularly of MGDG and DGDG.

In order to test if a significant amount of TAG is produced in *D. tertiolecta* from degradation of pre-formed PLs, we pre-labeled cell cultures with [^14^C]PlA before the onset of N deprivation and measured the transfer of ^14^C from PLs into TAG during the progress of N deprivation. After 2 h of labeling with [^14^C]PlA, which labels >90% of maximum PLs (Supplementary Fig. S2), cells were washed and transferred to N-deprived media for 8 d. Each day, samples were removed, lipids were extracted and separated, and the level of ^14^C in PLs and in TAG was determined.

**Fig. 8.**
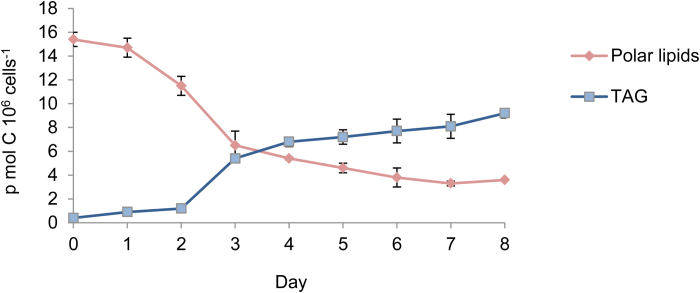
Formation of TAG from pre-formed PLs. *Dunaliella tertiolecta* cells were labeled for 2 h with [^14^C]PlA in complete growth medium. The cells were washed and transferred to fresh N-deprived medium. Each day samples were fractionated and analyzed as described in the Materials and methods. Results are expressed in pmol PlA incorporated per day per 10^6^ cells (means ± SD of three independent experiments). (This figure is available in colour at *JXB* online.)

Analysis of the levels of ^14^C in PLs and in TAG shows a progressive decrease in ^14^C level in PLs and a parallel increase in level in TAG (Fig. 8). From the onset to the eighth day of N deprivation, the ^14^C contents in PLs decreased by 12 pmol 10^6^ cells^−1^ whereas in TAG they increased by 9 pmol 10^6^ cells^−1^, indicating a conversion of 75% of the PlA from pre-formed PLs into TAG. Most of the conversion took place during the third day of N deprivation. If we assume that the transfer of other FAs from PLs into TAG is similar to that of PlA, this would mean that 75% of the FAs in pre-formed PLs are converted into TAG.

Quantification of the actual level of TAG produced from pre-formed PLs is difficult to estimate because we do not know if the pre-labeling with PlA leads to a homogenous labeling of all PLs that are being utilized for formation of TAG and also we do not know the exact internal concentration of native PlA within the cells, which may significantly dilute the added [^14^C]PlA.

The 75% conversion of FAs in pre-formed PLs into TAG is surprisingly high. By assuming a homogenous labeling of all PLs in the cells during the 2 h incubation with PlA, this would imply conversion of 130 nmol C 10^6^ cells^−1^ (from a total PL level of 169 nmol C 10^6^ cells^−1^; Supplementary Table S1), which is not realistic because it is far higher that the total decrease in the level of PLs during N deprivation (55 nmol C 10^6^ cells^−1^; Supplementary Table S1). This discrepancy suggests that only a small fraction of the total PLs in the cells are turning over and are being labeled with [^14^C]PlA during the 2 h incubation period, and that TAG is preferentially produced from this turning-over PL fraction.

## Discussion

**Scheme 1.**
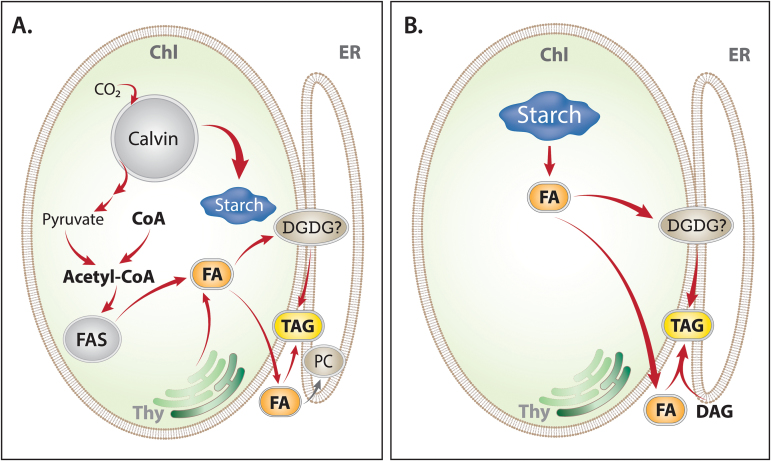
Proposed metabolic adaptation to N deprivation involved in TAG biosynthesis in *D. tertiolecta.* (A) Early stages of N deprivation; (B) late stages of N deprivation. Up-regulated reactions are indicated by red arrows. Enzymes are written in italics. PM, plasma membrane; ER, endoplasmatic reticulum; CE, chloroplast envelope; Thy, thylakoids.

The results presented in this work suggest that *D. tertiolecta* responds to N deprivation in two distinct metabolic phases: on the first day, cells still continue to divide, albeit at a progressively slower rate, photosynthetic CO_2_ assimilation activity drops, and the cells accumulate massive levels of starch in the chloroplast, accounting for over two-thirds of the total assimilated carbon. In the second phase, starting on the second day of N deprivation, cell division almost stops, photosynthesis drops further to 5% of that in control cells, the starch level reaches a steady state, and TAG is produced, mostly by recycling of starch carbon (Schemes 1 A, B). So the general carbon metabolism switched from photosynthetic carbon assimilation to starch degradation and the carbon reserves gradually change from all starch to progressively increasing TAG levels. A similar biphasic response to N deprivation with respect to starch and TAG biosynthesis has been recently demonstrated by several transcriptomic and metabolomic studies carried out in *Chlamydomonas* (Balby *et al.*, 2013; Goodenough *et al.*, 2014; Schmollinger *et al.*, 2014; Park *et al.*, 2015).

The surprising finding that most TAG produced during N deprivation is made from starch is novel and, to the best of our knowledge, these studies provide the first direct demonstration of carbon allocation from starch into TAG during N deprivation in algae. However, the idea that starch carbon is mobilized into TAG is consistent with reports in several recent studies.

For example, a recent transcriptomic analysis of metabolic changes during N deprivation in *D. tertiolecta* (Tan *et al.*, 2016) demonstrated activation of several enzymes potentially involved in starch to TAG mobilization: starch phosphorylase and phosphofructokinase that are involved in starch degradation; the chloroplast envelope triose phosphate transporter and glycerol phosphate dehydrogenase involved in the supply of glycerol phosphate needed for TAG formation; and of pyruvate kinase and pyruvate dehydrogenase that are involved in formation of acetyl-CoA, the key precursor metabolite in FA biosynthesis. All these enzymes could potentially take part in the mobilization of chloroplastic starch carbon into TAG.

In contrast, most FA biosynthesis genes, such as acetyl-CoA carboxylase (ACCase) and ketoacyl-ACP-synthase-I (KASI), were found to be either unaffected or down-regulated during N deprivation. Similar results have been previously described in transcriptomic analyses in *Nannochloropsis* sp. and in *Chlorella vulgaris* (Dong *et al.*, 2013; Li *et al.*, 2014; Fan *et al.*, 2015), which accumulate higher levels of TAG than *D. tertiolecta*. The reason for these observations is not clear. One explanation that has been proposed is that under N deprivation most FAs are supplied from degradation of PLs, particularly in the chloroplast membrane. This may be the case in *Nannochloropsis* and in *C. vulgaris* but, according to the present results, is not the case in *D. tertiolecta*. Another possibility would be that the steady-state activity of key FA biosynthetic enzymes is already high and thus not limiting under N-deficient conditions. In that case, FA synthesis would most probably be dependent on substrate availability, such as acetyl-CoA, as recently proposed (Avidan *et al.*, 2015). The degradation of starch and the corresponding increase of TAG in *D. tertiolecta* is consistent with that idea. A third possibility may be that the biosynthesis of FA from starch involves activation of an alternative biosynthetic pathway that substitutes the chloroplast constitutive FAS pathway, which has not yet been identified.

The finding that *D. tertiolecta* utilizes starch as the major carbon source for TAG biosynthesis is also consistent with the finding that several starch-less mutants of *Chlamydomonas* do not exhibit an increase in TAG accumulation under N deprivation which has been previously observed in other starch-less mutants strains of *Chlamydomonas* and of *Chlorella* (Hirano *et al.*, 1997; Ramazanov and Ramazanov, 2006; Wang *et al.*, 2009; Work *et al.*, 2010; Siaut *et al.*, 2011). This finding is in line with the idea that starch serves as a major carbon source for production of TAG; hence, impairing starch accumulation may also impair the accumulation of TAG.

In fact, our calculated levels of TAG formation from starch may be underestimated because we did not measure the possible contribution of starch made before the onset of N deprivation that amounts to 40% of the total starch in the cells (Supplementary Table S1).

The contribution of other cellular components, such as proteins, whose levels decrease during N deprivation (Supplementary Table S1), for the formation of TAG during N deprivation, cannot be excluded. However, it seems to be negligible, first because during N deprivation there is also a significant biosynthesis of new proteins (Fan *et al.*, 2011; Boyle *et al.*, 2012; Balby *et al.*, 2013; Dong *et al.*, 2013; Li *et al.*, 2014; Recht *et al.*, 2014; Schmollinger *et al.*, 2014; Park *et al.*, 2015; Shin *et al.*, 2015; Longworth *et al.*, 2016), and it may be expected that amino acids released during protein degradation will be first utilized for biosynthesis of the newly induced proteins and also because the calculated sum of starch-derived and *de novo* made TAG closely fits the measured total TAG level (Fig. 6), leaving little room for additional major carbon sources.

The contribution of pre-formed PLs to the production of TAG is difficult to estimate because the exact internal concentration of PlA is not known and also because the labeling of different PLs with PlA is not homogenous, as discussed in the Results. An attempt to estimate internal PlA concentration roughly in the cells from isotope dilution (Table S3 and supplementary comments) yielded a value of ~1 μM internal PlA. Calculation of the level of TAG produced from pre-formed PLs by assuming an internal concentration of 1 μM PlA and homogenous labeling yields a value of <5 nmol C 10^6^ cells^−1^. This value is negligible in comparison with the levels of TAG produced from starch and *de novo*. Also the close correlation between the calculated levels of the starch made plus *de novo* made TAG with the total TAG (Fig. 6) leaves little room for TAG made from pre-formed PLs, and suggests that this may be a minor contributor to TAG production in *D. tertiolecta*.

The minor contribution of pre-formed PLs to TAG biosynthesis most probably results from degradation of the chloroplast galactoglycerolipids MGDG, DGDG, and SQDG, which in *Dunaliella* takes place during the first 2 d of N deprivation (Davidi *et al.*, 2014). These results are in line with several recent studies showing that PLs in algae and in plants, in particular the chloroplast major galactoglycerolipids MGDG and DGDG, but also extra-chloroplast membrane lipids such as diacylglyceryltrimethylhomoserine (DGTS), serve as a source of FAs for synthesis of TAG during N deprivation (Joyard *et al.*, 2010; Boyle *et al.*, 2012; Li *et al.*, 2012; Yoon *et al.*, 2012; Goncalves *et al.*, 2013; Kim *et al.*, 2013; Sanjaya *et al.*, 2013; Simionato *et al.*, 2013; Li *et al.*, 2014). The major candidates to perform this FA release and transacylation are PESs. These enzymes are up-regulated under N deprivation and catalyze the hydrolysis and transacylation of FAs from chloroplast membrane galactoglycerolipids into TAG (Lippold *et al.*, 2012). PES-like proteins are also up-regulated under N deprivation in *Dunaliella* (Davidi *et al.*, 2015).

The transfer of FAs from PLs formed during the first days of N deprivation into TAG (Fig. 5B) suggests that PLs function as intermediates in acyl transfer into TAG. This acyl transfer is spread over 5–7 d and amounts to 20–25% of the total PlA in TAG. The rates of acyl transfer into TAG may be much higher since it is likely that some of the FAs produced from starch degradation are also incorporated into TAG via this pathway.

The identity of these PLs is not clear, but we have several indications that it may be DGDG. The significant *de novo* synthesis of this galactoglycerolipid under N deprivation (Fig. 7) suggests enhanced turnover of this major chloroplast membrane galactoglycerolipid under N deprivation. In a subsequent work, we will report further evidence obtained from a time-course lipidome analysis that this particular galactoglycerolipid undergoes remarkable turnover under N deprivation in green algae (O. Avidan *et al*., unpublished results). It is possible, therefore, that in *D. tertiolecta*, some of the FAs that are utilized for TAG biosynthesis under N deprivation are first incorporated into DGDG, similar to the role of PC acyl editing in N-containing medium. This finding is in line with previous pulse labeling experiments in *C. reinhardtii* (Li *et al.*, 2012).

The preference to produce TAG from starch carbon in Dunaliella suggests that it has some metabolic advantage under N limitation. In most plants and green algae, starch is accumulated during the day and consumed during the night in an almost linear manner (Stitt and Zeeman, 2012). Influenced and controlled by the circadian clock, starch synthesis and degradation cycles are also observed under constant light regimes in both plants and algae (Lu *et al.*, 2005; Siaut *et al.*, 2011; Avidan *et al.*, 2015; Chen *et al.*, 2015). However, when N becomes limiting, growth is almost immediately restricted while photoassimilates and reducing equivalents are accumulated and channeled towards not only starch, but TAG synthesis as well. Indeed, starch is a more flexible carbon reserve than TAG, and its conversion into metabolic building blocks such as sugar-phosphates and amino acids is faster and therefore affords an advantage for fast recovery from short stress periods. In contrast, TAG contains about twice the metabolic energy and carbon level as starch and is also a better sink for excess redox energy which poses a risk for survival, and therefore may be favored in long-term stress periods. Considering the fact that most algae accumulate both starch and TAG, the efficiency of starch synthesis and interconversion into TAG should be taken into account in future attempts to manipulate TAG levels in microalgae. It will be interesting to learn if other algae species, such as those that accumulate high levels of TAG and low levels of starch, also utilize starch as an intermediate carbon pool for biosynthesis of TAG.

In summary, our results suggest that around two-thirds of the total TAG produced during N deprivation in *D. tertiolecta* is made from starch, one-third is made *de novo* mostly by transfer via PLs, and a very small amount may be contributed by degradation of pre-formed PLs.

## Supplementary data

Supplementary data are available at *JXB* online.

Fig. S1. The separation of neutral and polar lipids by TLC.

Fig. S2. The incorporation of [^14^C]palmitic acid into lipids and into solvent-insoluble cellular components in *D. tertiolecta*.

Table S1. The total ^14^C carbon fractionation into different cellular components during the progress of N deprivation

Table S3. [^14^C]PlA incorporation levels into PLs and into TAG in cells cultured in control or in N-deprived media, and supplementary information about palmitic acid in *D. tertiolecta*.

## Supplementary Material

supplementary_Figures_S1-S2 and Tables_S1-S3

## Abbreviations:

ACP: acyl-carrier protein
DGDG: digalactosyldiacylglycerol
FA: fatty acid
FAS: fatty acid synthesis
MGDG: monogalactosyldiacylglycerol
PC: phosphatidylcholine
PES: phytylester synthase
PL: polar lipid
PlA: palmitic acid
TAG: triacylglycerol.
