# Supplementary material for: Triacylglycerol is produced from starch and polar lipids in the green alga Dunaliella tertiolecta
Source: J Exp Bot. 2017 Sep 11;68(17):4939–50. doi: 10.1093/jxb/erx280 (PMC5853294; doi:10.1093/jxb/erx280)
Supplement: supplementary_Figures_S1-S2 and Tables_S1-S3 [file erx280_suppl_supplementary_figures_s1-s2_tables_s1-s3.pdf]

Fig. S1

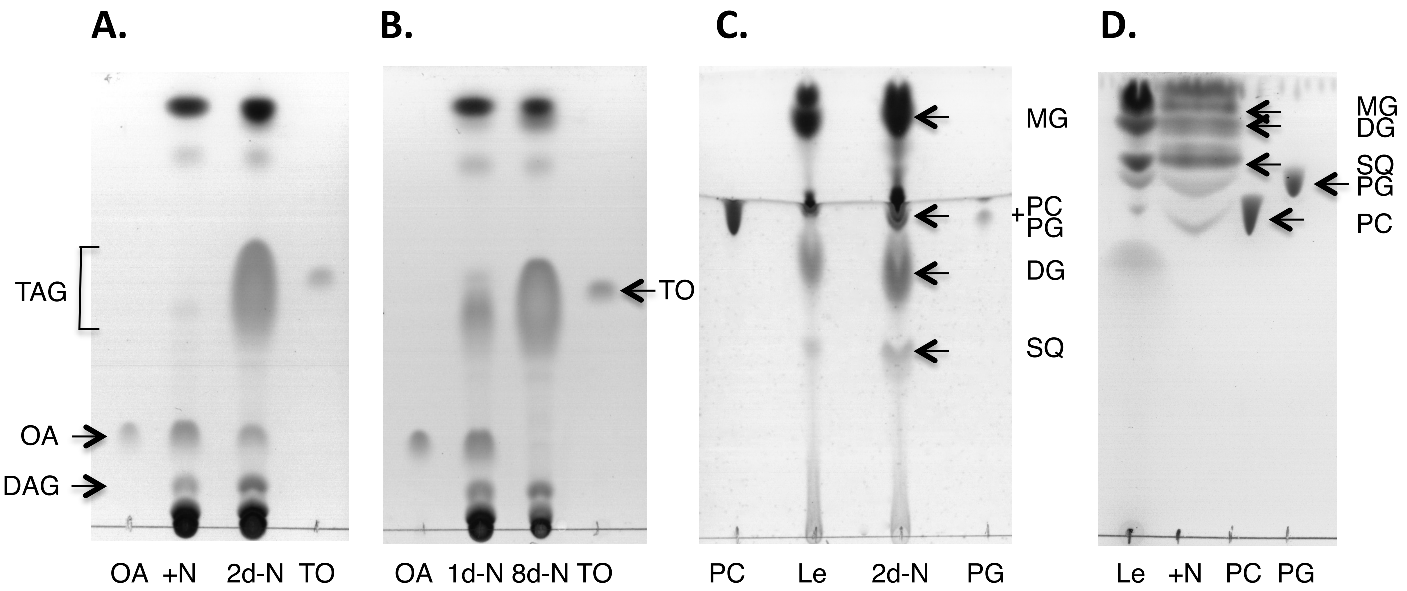

Fig. S1. TLC analysis of neutral (A,B) and of polar (C,D) lipids. The TLC plates were developed in: **(A,B)** *n*-hexane: diethyl-ether:acetic acid (85:22:1), **(C)** chloroform: methanol: acetic acid:water (90:20:12:4); **(D)** chloroform: methanol: water (60:30:4). +N, 1d-N, 2d-N, 8d-N- lipid extracts of *D. tertiolecta* cultured in N sufficient medium or deprived of nitrogen for 1d, 2d or 8d, respectively. Le-lettuce thylakoids lipid extracts. OA-oleic acid, TO- triolein, DAG- diacylglycerol, PC- phosphatidylcholine, PG- phosphatidylglycerol, MG- monogalactosyldiacylglycerol, DG- digalactosyldiacylglycerol, SQ- sulphoquinovosyldiacylglycerol.

Fig. S2

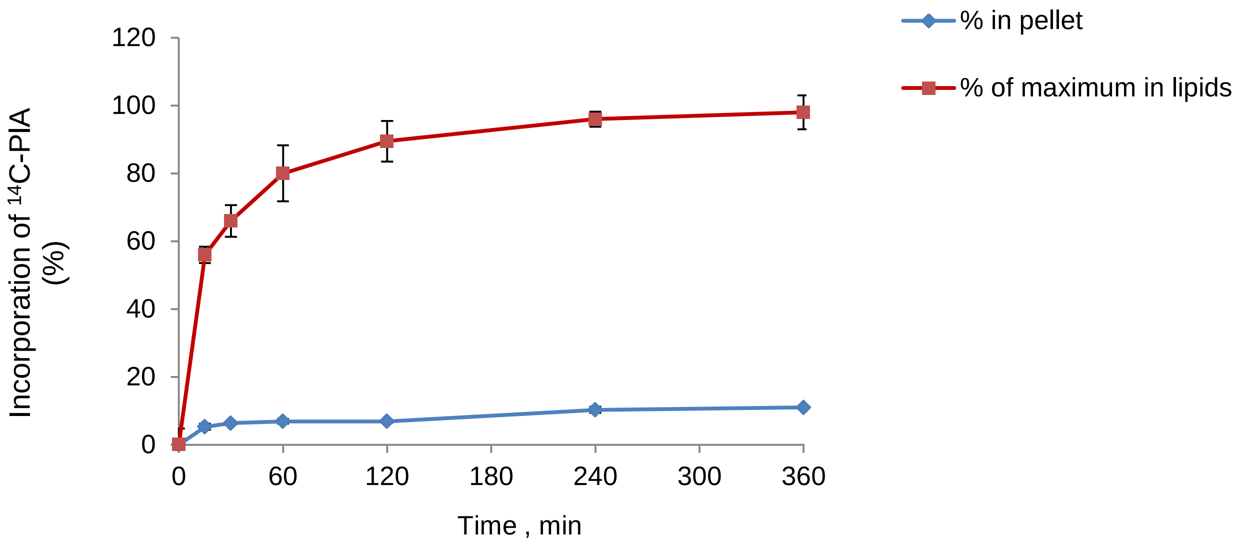

Fig. S2. Incorporation of ^{14}C-PlA into lipids and into solvent-insoluble cellular components in *D. tertiolecta*. *D. tertiolecta* in complete (+N) growth medium was labeled with 0.1 µCi ^{14}C-PlA/10 ml. After the indicated times, samples were removed, washed once with 100 µM PlA and once with 1% BSA, the ^{14}C contents in the lipid extract and in the extracted cell pellets, that contain mostly starch and proteins, were determined as described under Materials and methods.

Table S1. Total ^{14}C carbon fractionation during N deprivation

A. In n mol C 10^6 cells^{-1}

Time [h]	Total C	Starch	Water-Soluble	Protein	Polar L.	TAG	Recovery %
0	1,769±123	765±85	245±30	370±26	169±15	4±0.2	82.1
6	2,295±212	1,180±65	225±20	350±12	165±21	6±0.5	79.2
12	2,454±190	1,500±110	220±20	345±33	162±9	16±0.5	91.4
24	2,737±205	1,710±90	210±15	330±21	160±12	42±2	89.6
48	3,041±135	1,890±190	205±22	310±10	151±6	108±3	87.6
96	2,959±212	1,870±130	190±17	275±16	125±16	230±6	91.0
144	2,947±200	1,860±240	200±10	248±18	120±3	302±8	92.6
192	2,941±155	1,850±110	185±15	230±9	115±5	346±12	92.7

B. In % of total C

Time [h]	Starch, %	Water-soluble %	Protein %	Polar lipids %	TAG %
0	43.2	13.8	20.9	9.6	0
6	51.4	9.8	15.2	7.2	0
12	61.1	9.0	14.1	6.6	0
24	62.5	7.7	12.1	5.8	1.5
48	62.1	6.7	10.2	5.0	3.6
96	63.2	6.4	9.3	4.2	7.8
144	63.1	6.8	8.4	4.1	10.2
192	62.9	6.3	7.8	3.9	11.8

Cells were pre-labeled with ^{14}C-bicarbonate in complete growth medium and then transferred to N-deficient medium (time 0) containing the same ^{14}C-bicarbonate levels as described in Materials and Methods and in Fig. 2A. Cell fractionation and determination of total and fractional carbon contents in each cell fraction were performed as described under Materials and methods (± represent standard deviation).

Table S2. Comparisons of the increase in levels of starch and of TAG during N deprivation by different methods.

Medium	Starch		TAG	
	^{14}C-BC	Iodine	^{14}C-BC	GC
+N	23.1 ± 2.6	20 ± 6	0.08 ± 0.004	0.3 ± 0.15
-N	55.5 ± 3.3	70 ± 16	5.6 ± 0.24	9.4 ± 1.8

All values in the table are in units of µg/10^6 cells. The values for ^{14}C bicarbonate incorporation (^{14}C-BC) for starch and TAG were calculated from the values shown in Table S1. Starch determination by the iodine method was performed as previously described (Avidan et al., 2015). TAG determination by gas chromatography FA analysis was performed by lipid extraction, TAG isolation on thin-layer chromatography plates as previously described (Davidi et al., 2014) and the fatty acid contents was determined by Prof. I. Khozin-Goldberg at The Negev University as previously described (Khozin-Goldberg et al., 2005) and used to calculate the TAG contents. +N: control cells cultured in complete growth medium for 48 h with 50 mM Na-^{14}C-bicarbonate; -N: cells cultured for additional 8 days without N at the same ^{14}C-bicarbonate concentration (± represent standard deviation).

Table S3: ^{14}C-PlA incorporation levels into PL and into TAG in cells cultured in control or in N-deprived media.

Culture	PL	TAG	Total incorp.
+N	12.2 ± 2.3	0.4 ± 0.2	18.5 ± 2.8
-N	7.4 ± 1.0	11.6 ± 2.4	23.1 ± 3.6

D. tertiolecta cells cultured for 24 h in complete (+N) growth medium or for 48 h in N-deprived (-N) medium were labeled for 24 h with ^{14}C-PlA. Lipids were extracted and separated on TLC plates as in Fig. S1A, the lipid bands corresponding to PL (two major bands near the origin at Fig. S1A) and TAG were cut, extracted and counted. Total incorporation values represent the total ^{14}C counts in washed cell pellets. Values in the table are in units of p mol PlA 10^6 cells^{-1} and represent 3 independent experiments.

Further comments concerning the labeling of *D. tertiolecta* with ^{14}C-PlA.

Labeling of *D. tertiolecta* with PlA will be described and discussed in detail in a subsequent publication. Some experimental details that are relevant to the present study are described in brief below.

1. Effect of PlA on growth and physiological parameters: PlA does not inhibit growth rate, photosynthetic activity (oxygen evolution) or starch level up to a concentration of 100 µM. We found a slight increase in the level TAG produced in the presence of 10 µM-100 µM (10%-15%).
2. PlA turnover: The incorporation of PlA into most polar lipid components in *D. tertiolecta* including the FA pool is fast and reaches a steady-state within 10 min or less. Therefore we concluded that the incorporation rate of PlA into TAG is not limited by diffusion barriers or by internal pools.
3. We attempted to estimate the internal concentration of PlA in the cells by dilution of the ^{14}C-PlA with different concentrations unlabeled PlA. We assumed that an external concentration equal to the endogenous concentration in the cells will inhibit ^{14}C-PlA incorporation by about 50%. We found that 1 µM PlA inhibited the incorporation into TAG by 50%. According to this criteria, the internal concentration of PlA in the cells is around 1 µM, and

since we label with 0.5 μM ^{14}C-PlA we expect an isotope dilution of around 3-fold inside the cells.
